# Structural Studies of *Klebsiella pneumoniae* Fosfomycin-Resistance Protein and Its Application for the Development of an Optical Biosensor for Fosfomycin Determination

**DOI:** 10.3390/ijms25010085

**Published:** 2023-12-20

**Authors:** Christina Varotsou, Farid Ataya, Anastassios C. Papageorgiou, Nikolaos E. Labrou

**Affiliations:** 1 Laboratory of Enzyme Technology, School of Applied Biology and Biotechnology, Agricultural University of Athens, 75 Iera Odos Street, GR-11855 Athens, Greece; chrisvar@aua.gr; 2 Department of Biochemistry, College of Science, King Saud University, P.O. Box 2455, Riyadh 11451, Saudi Arabia; fataya@ksu.edu.sa; 3 Turku Bioscience Centre, University of Turku and Åbo Akademi University, 20521 Turku, Finland; anapap@utu.fi

**Keywords:** antibiotic resistance, fosfomycin-resistance protein, *Klebsiella pneumoniae*, *Pseudomonas aeruginosa*, glutathione, fosfomycin, metalloenzyme, biosensor

## Abstract

Fosfomycin-resistance proteins (FosAs) are dimeric metal-dependent glutathione transferases that conjugate the antibiotic fosfomycin (Fos) to the tripeptide glutathione (γ-Glu-Cys-Gly, GSH), rendering it inactive. In the present study, we reported a comparative analysis of the functional features of two FosAs from *Pseudomonas aeruginosa* (FosAPA) and *Klebsiella pneumoniae* (FosAKP). The coding sequences of the enzymes were cloned into a T7 expression vector, and soluble active enzymes were expressed in *E. coli*. FosAKP displayed higher activity and was selected for further studies. The crystal structure of the dimeric FosAKP was determined via X-ray crystallography at 1.48 Å resolution. Fos and tartrate (Tar) were found bound in the active site of the first and second molecules of the dimer, respectively. The binding of Tar to the active site caused slight rearrangements in the structure and dynamics of the enzyme, acting as a weak inhibitor of Fos binding. Differential scanning fluorimetry (DSF) was used to measure the thermal stability of FosAKP under different conditions, allowing for the selection of a suitable buffer to maximize enzyme operational stability. FosAKP displays absolute specificity towards Fos; therefore, this enzyme was exploited for the development of an enzyme-based colorimetric biosensor. FosAKP was tethered at the bottom of a plastic cuvette using glutaraldehyde chemistry to develop a simple colorimetric method for the determination of Fos in drinking water and animal plasma.

## 1. Introduction

Antimicrobial resistance poses a great threat to modern societies, since it is responsible for at least 700,000 deaths per year globally [1,2]. The increase in the number of resistant bacterial cases, together with the relatively slow development rates of new antimicrobial drugs, has led to the reassessment and reintroduction of old antibiotics. The broad-spectrum antibiotic fosfomycin ((1R,2S)-epoxypropylphosphonic acid (Fos)) is synthesized by certain *Streptomyces* and *Pseudomonas* strains [3]. Fos is effective against both Gram-positive and Gram-negative pathogens by inhibiting the first committed step in cell wall biosynthesis. Administration of Fos alone or in combination with other antibiotics has shown bactericidal activity against several multidrug-resistant strains (MDR strains) [4,5,6].

**Figure 1:**
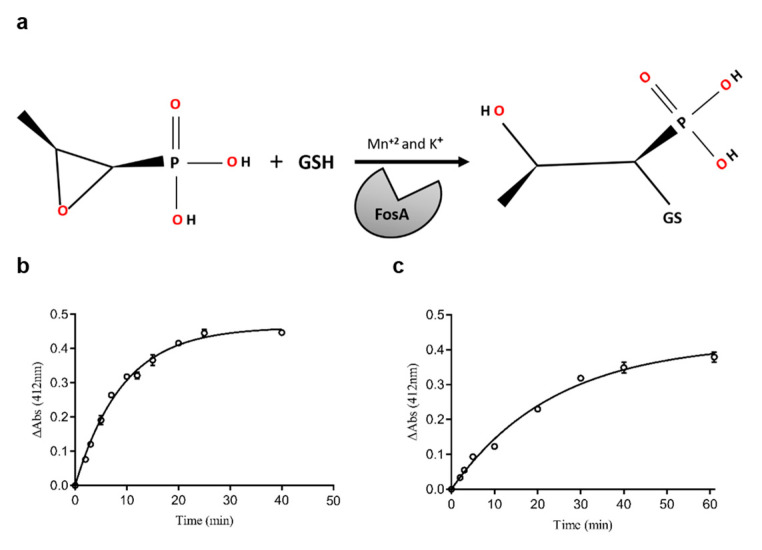
(**a**) The reaction catalyzed by FosA. This enzyme cleaves the epoxide ring of Fos by conjugating GSH to carbon 1 (C1) of the epoxide moiety of the antibiotic. This enzyme requires both Mn^2+^ and K^+^ for displaying optimal catalytic activity. (**b**) Time course of the Fos conjugation with GSH catalyzed by FosAPA. (**c**) Time course of the Fos conjugation with GSH catalyzed by FosAKP. The absorbance change (ΔAbs) was measured at 412 nm. The one-phase association curve was fitted to the dataset using GraphPad Prism 7.00. Data are reported as the mean ± standard error of three replicates. R^2^ = 0.9925 for (**b**) and R^2^ = 0.9916 for (**c**).

Three different mechanisms have been reported that lead to Fos-resistant bacterial strains. These mechanisms are: (i) mutations in the uptake and transport systems of antibiotics, (ii) mutations in the Fos target UDP-N-acetylglucosamine enolpyruvyl transferase (MurA), and (iii) drug modifications attributed to the presence of one of four different types of Fos-resistance proteins (FosA, FosB, FosC, or FosX) [4,7,8,9,10]. Fos-resistance proteins are metalloenzymes that inactivate Fos through the addition of nucleophiles, such as water, GSH, L-cysteine, and bacillithiol (BSH), to the oxirane ring of the molecule. The adduct formed via this enzymatic reaction displays no bactericidal activity. Two types of thiol transferase enzymes (FosA and FosB) have been identified in the genomes of microorganisms. The most well-characterized Fos-resistance enzyme is FosA, which is responsible for most cases of Fos-resistant, Gram-negative bacteria, including *Escherichia coli* [2,11,12]. FosA enzymes are manganese (Mn^+2^)- and potassium (K^+^)-dependent glutathione transferases that conjugate GSH to carbon 1 (C1) of Fos (Figure 1a), leading to the opening of the epoxide ring of Fos. FosB is a Mg^2+^-dependent L-cysteine thiol transferase.

FosA enzymes share low sequence and structural similarities with the members of the cytosolic GST family. They belong to the vicinal oxygen chelate (VOC) superfamily of enzymes. Members of the VOC family are structurally related; however, they catalyze a diverse set of metal-dependent reactions [13]. For example, members of this family include extradiol dioxygenases that participate in the meta-cleavage of a variety of hydroxylated aromatic compounds, glyoxalases I that catalyze the isomerization of hemithioacetals, and methylmalonyl-CoA epimerases involved in the epimerization of (2S)-methylmalonyl-CoA to its (2R)-stereoisomer [14,15]. Members of the VOC family share a common fold composed of two βαβββ units that form an incompletely closed β-sheet barrel [13].

The catalytically active form of the FosA enzyme is a homodimer, whose tertiary structure complies with the fold of members of the VOC superfamily of enzymes [16]. FosA utilizes both manganese and potassium ions to catalyze its enzymatic reaction. The manganese ion appears to bind to the Fos molecule and acts as an electrophilic catalyst during the addition of GSH to the antibiotic. The potassium ion (K^+^) binds to the K^+^-loop located close to the active site of the enzyme, resulting in an approximately 100-fold increase in the catalytic reaction [2,17,18].

Fos is considered a safe antibiotic for human use, causing fewer side effects and being administered in a single dose compared to other antibiotics [3,19]. Fos is a re-emerging bactericidal antibiotic and has attracted renewed interest for the treatment of serious systemic infections caused by multidrug-resistant *Enterobacteriaceae*. Fos displays a high ability to penetrate tissues; therefore, it is recommended against infections of the CNS, soft tissues, bones, and lungs, and the treatment of abscesses. Recently, its clinical use has gained significant attention owing to the dramatic increase in the number of infections caused by MDR bacteria worldwide [3,20]. This fact urges the development of a rapid, simple, and specific quantification method for Fos for further clinical studies. The development of a rapid and simple quantification method for Fos can provide insights into the pharmacokinetics and pharmacodynamics (PK/PD) of Fos, which is essential for optimizing its treatment and preventing the development of resistance. In addition, since important pharmacological data and the understanding of Fos-resistant mechanisms are scarce and/or controversial at present, additional PK/PD studies are urgently needed.

Enzymes are environmentally friendly, cost effective, and efficient analytical tools compared to common chemical reagents [21,22]. Thus, their use in biomedical analyses has increased in recent decades. However, their routine use is frequently restricted by their low operational stability. Enzyme-based biosensors are generally preferred over free enzymes, as they allow real-time monitoring, signal stability, long shelf-life, and high reproducibility [21,23,24,25].

In the present study, two FosA isoenzymes, FosAPA and FosAKP, from *Pseudomonas aeruginosa* and *Klebsiella pneumoniae*, respectively, were expressed in *Escherichia coli* cells, and their structural and catalytic properties were investigated. The comparative study of two FosA isoenzymes allowed for the selection of FosAKP as the more suitable enzyme, in terms of specificity, specific activity, and kinetic parameters for the development of an enzyme-based biosensor for Fos determination. For this requirement, FosAKP was tethered by cross-linking with glutaraldehyde and bovine serum albumin (BSA), and the biocatalytic material was used to develop a rapid photometric assay for the determination of Fos.

To the best of our knowledge, our work reports the first enzyme-based biosensor for Fos. The majority of published analytical methods for Fos have mainly focused on LC-MS/MS, HPLC, or HPLC-MS/MS [26,27,28].

## 2. Results and Discussion

### 2.1. The Expression, Purification, and Substrate Specificity of FosAPA and FosAKP

Recombinant FosAPA and FosAKP were overexpressed in *E. coli* BL21(DE3)pLysS cells and purified using Ni-IDA-Sepharose affinity chromatography. The purity of the isoenzymes was analyzed using SDS-PAGE (Supplementary Figures S1 and S2), and the results showed that, in both cases, an intense protein band, corresponding to an approximately 16 kDa molecular mass, was purified. Both purified proteins were catalytically active and were able to catalyze the conjugation of GSH to Fos, resulting in the opening of the epoxide ring. Figure 1b,c show the time course of Fos conjugation with GSH, catalyzed by FosAPA and FosAKP, respectively. The amino acid sequences of FosAPA and FosAKP share 65.73% identity; however, their specific activity (SA, U/mg protein) differs significantly. The SAs of FosAPA and FosAKP were measured as 66.7 U/mg and 147.3 U/mg, respectively, showing that FosAKP exhibits > 2-fold higher SA compared to FosAPA. These results agree with the available data in the literature, which support the finding that FosAKP is a more catalytically active enzyme than FosAPA [2].

An important catalytic feature of FosAKP is its absolute substrate specificity for Fos [2]. We further assessed the substrate specificity of FosAKP and FosAPA using a range of electrophilic compounds commonly used as glutathione transferase (GST) substrates (Supplementary Table S1). The results showed that FosAKP is catalytically inactive towards all model GST substrates, confirming the absolute specificity of this enzyme. Considering its high catalytic activity and absolute substrate specificity, FosAKP it was selected for further studies.

### 2.2. Structural Analysis

**Figure 2:**
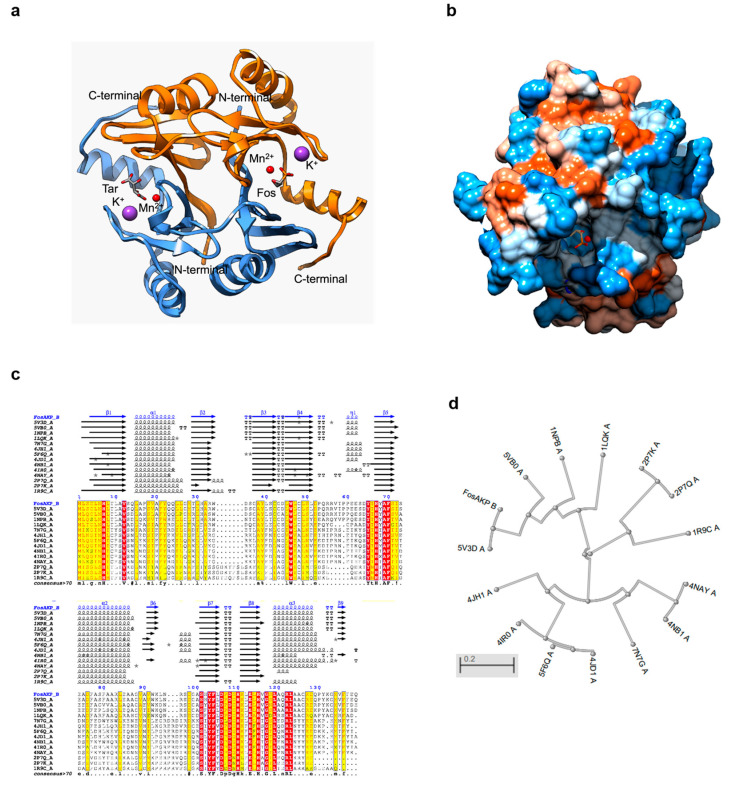
Crystal structure of the FosAKP homodimer. (**a**) Ribbon representation of FosAKP with bound Tar (chain A), Fos (chain B), K^+^ (magenta sphere), and Mn^2+^ (red sphere). Chain A is colored pale blue and chain B is colored orange. (**b**) Hydrophobicity surface of FosAKP with bound Fos and Mn^2+^. Fos is represented as sticks and are colored according to the atom type. Bound Mn^2+^ is represented as red spheres. The structure is colored from blue for the most hydrophilic to white to orange/red for the most hydrophobic. (**c**) Structural alignments of FosA homologues performed via ENDscript analysis [29]. The PDB codes of homologue enzymes are depicted. Helixes are shown as squiggles, β-strands are depicted as arrows and five-pointed star represents contacts between protein residues and automatically or manually kept hetero-compounds. (**d**) Phylogenetic analysis. The phylogenetic tree was constructed using the NCBI tree viewer, based on sequence alignments (Clustal O) [30] performed via ENDscript analysis. The PDB codes of homologue enzymes are depicted.

The crystal structure of FosAKP was determined via X-ray crystallography and was resolved at 1.48 Å resolution (Table 1). The resolution of the structure achieved in the present work was comparable and slightly better to that previously reported in the literature (1.54 Å; PDB accession number: 5V3D; by Klontzt et al., 2017) [2]. Structure-based superposition of the two structures gave a root-mean-square deviation of 0.18 Å for 137 Cα pairs, suggesting subtle differences between them. The three-dimensional structure of FosAKP (Figure 2a) is folded into a dimer, providing an active site per monomer. Both subunits are composed of two tandem βαβββ motifs that follow a 3D-domain swapping during homodimer formation. Consequently, each active site was formed by residues originating from both subunits. Each active site is mainly composed of polar residues, forming a hydrophilic cavity (Figure 2b). Structural alignments of FosA-homologous enzymes revealed a conserved overall structure with several strictly conserved amino acid residues (Figure 2c,d) [29,30].

**Figure 3:**
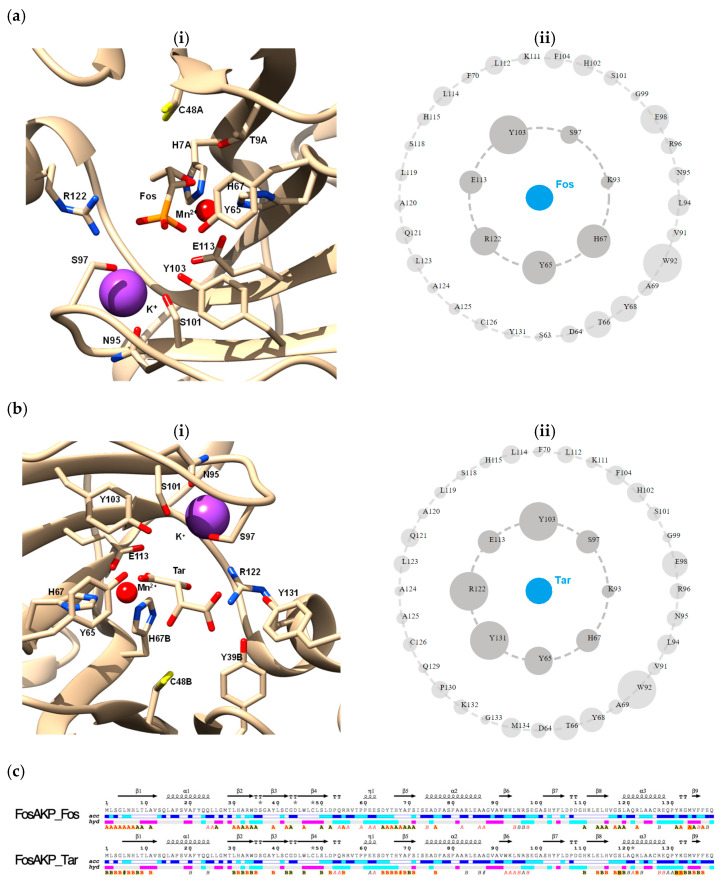

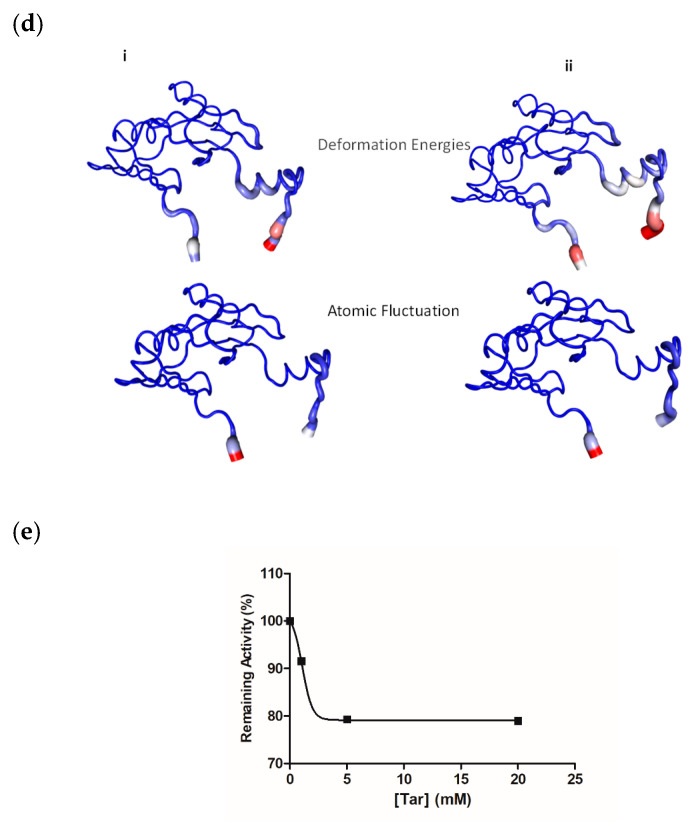
(**a**). (**i**) Key amino acid residues that contribute to Fos, Mn^2+^, and K^+^ binding are shown in sticks and colored according to their atom type. Bound Mn^2+^ and K^+^ are represented as red and magenta spheres, respectively. (**ii**) Asteroid plot of interactions of Fos. The inner ring indicates the first shell of immediate atomic contacts. The outer ring indicates the second shell of extended atomic contacts. The size of the circle is proportional to the total number of contacts the residue is involved in with any of the residues in the ring inwards to it. (**b**). (**i**) Important amino acid residues for Tar binding are shown in sticks and colored according to their atom type. Bound Mn^2+^ and K^+^ are represented as red and magenta spheres, respectively. (**ii**) Asteroid plot of interactions of Tar. The inner ring indicates the first shell of immediate atomic contacts. The outer ring indicates the second shell of extended atomic contacts. The size of the circle is proportional to the total number of contacts the residue is involved in with any of the residues in the ring inwards to it. (**c**). Solvent accessibility analysis with ENDscript [29]. Flat figure showing the sequence of FosAKP with secondary structure elements presented on top (helices with squiggles, β-strands with arrows, and turns with TT letters). Solvent accessibility is rendered by a first bar below the sequence (blue is accessible, cyan is intermediate, and white is buried) and hydropathy by a second bar below (pink is hydrophobic, white is neutral, and cyan is hydrophilic). Bottom letters and star symbols depict crystallographic protein: protein and protein: ligand contacts. A red letter (A for the FosAKP–Fos complex and B for the FosAKP–Tar complex) identifies a contact < 3.2 Å, while a black letter identifies a contact between 3.2 Å and 5 Å. The orange background signifies an amino acid involved in both a crystallographic and a non-crystallographic contact. (**d**). Plot of deformation energy and atomic fluctuation along the polypeptide chain of Fos-bound subunits (**i**) and Tar-bound subunits (**ii**). The analysis was conducted using the DynaMut web server [31]. The deformation/fluctuation magnitude is represented by thin to thick tubes colored blue (low) to red (high). Deformation energy provides a measure of local flexibility in the protein. Atomic fluctuations provide the amplitude of the absolute atomic motion. (**e**). The inhibition of FosAKP by Tar. The assays were performed in 0.1 M sodium dihydrogen phosphate buffer, pH 8, containing 100 mM KCl and 0.05 mM MnCl_2_ at 25 °C in the presence or the absence of different concentrations of Tar (0–20 mM).

A Fos molecule was found bound in the actives site of the first molecule of the dimer. In the active site of the second molecule, a tartrate molecule was bound, which originated from the crystallization buffer. Important residues that play crucial role in Fos binding include Ser97, Lys93, His67, Tyr65, Arg122, Glu113, and Tyr103 (Figure 3a). Mn^2+^ binding was coordinated by two His residues (His7 and His67) and Glu113. The binding of K^+^ was facilitated by a range of residues (namely Asn95, Ser97, Glu98, Gly99, Ser101, and His115) that form the so-called K^+^-loop [2].

Tar occupies the same location as Fos (Figure 3b). The majority of Fos-binding residues are also involved in Tar binding. However, two additional residues (Tyr131 from molecule A and Tyr39 from molecule B of the dimer) appeared to contribute significantly to Tar binding. The hydroxyl group of Tyr131 formed a hydrogen bond with the O1 atom of Tar (distance: 2.9 Å), whereas the O11 atom of Tar was in hydrogen bond distance (2.4 Å) with the hydroxyl group of Tyr39. Fos has 26 atomic contacts with its immediate amino acid neighbors, whereas Tar develops several additional atomic contacts, reaching 32 in total (Figure 3a(ii),b(ii)). The structures of Tar and Fos share a similar overall structure, polarity, size, and negatively charged groups (carboxylate vs. phosphonic groups). Therefore, it is most conceivable that Tar behaves as a substrate analogue and occupies the same site with the substrate. The structure of the homologue enzyme FosA from *Pseudomonas aeruginosa* in complex with phosphonoformate showed that phosphonoformate binds to the active site, acting as a minimal transition state analogue inhibitor [32].

The binding of Mn^2+^ and K^+^ to both subunits is coordinated by the same residues; therefore, both metals occupy identical locations in both subunits. Solvent accessibility analysis of the two subunits (Figure 3c) [29] revealed distinct differences at the C-terminal part between Gly99-Cys126. In particular, the C-terminal part of the Tar-bound subunit appears to be less solvent accessible (Figure 3c), suggesting the formation of a more compact complex.

The analysis of protein dynamics (Figure 3d) using normal mode analysis (DynaMut web server) [31] revealed several differences in the deformation energy along the polypeptide chain between the Fos-bound and Tar-bound subunits. The deformation energy provides a measure of the amount of local flexibility in a protein. Based on this analysis, it appeared that the C-terminal helix 3 of the Fos-bound subunit, which forms part of the substrate-binding site, displays higher deformation energy and therefore less flexibility. This observation supports the notion discussed above (Figure 3c) that FosAKP forms a more compact complex with Tar.

The binding of Tar to the active site suggested that Tar may act as an inhibitor of FosAKP. To test this hypothesis, kinetic assays were performed in the presence and absence of different Tar concentrations (0–20 mM); the results are shown in Figure 3e. Although Tar appeared to display moderate inhibition potency (i.e., 20.7% inhibition at 5 mM concentration in the assay mixture), it can be, however, considered as a lead scaffold for future drug design efforts.

### 2.3. Thermal Stability of FosAKP

**Figure 4:**
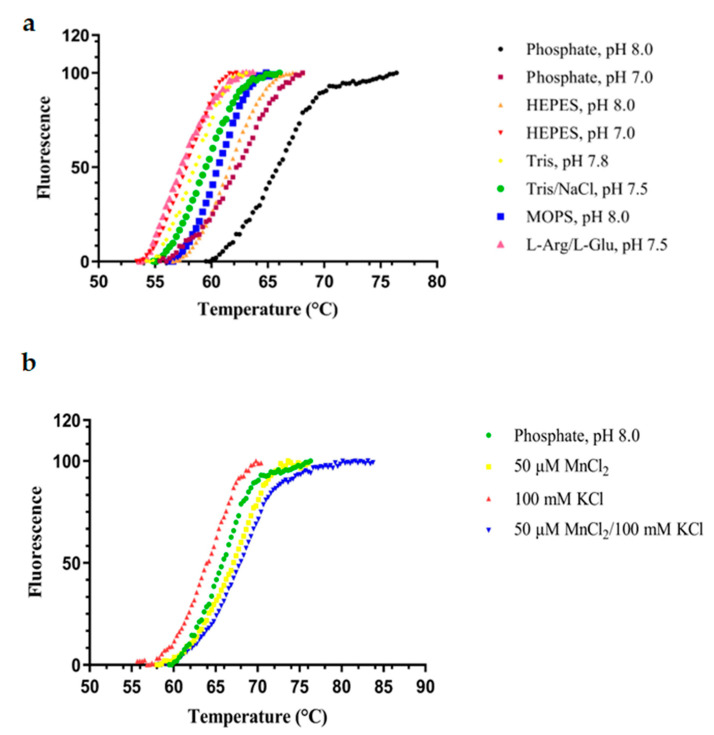
Normalized DSF data of the screening for the optimal buffer system for FosAKP. The melting temperature (T_m_) is the temperature at which a 50% denaturation of the protein occurs. It is defined as the inflection point of the melting curve. Data were fitted to the Boltzmann curve for T_m_ determination using GraphPad Prism 7.00 software. (**a**) Effect of different buffer systems on the melting temperature. (**b**) Effect of MnCl_2_, KCl, and MnCl_2_/KCl mixture on the melting temperature.

The stability of FosAKP under different buffer and pH conditions was investigated using differential scanning fluorimetry (DSF). Initially, the optimal buffer system, in which FosAKP exhibited its highest structural stability, was investigated. A range of different buffer systems and pH values was selected, and the melting temperature (T_m_) of the protein was determined (Figure 4). The buffers were selected based on their ability to provide buffering capacity in the range of 7–8, where the enzyme displays maximum activity. Also, the stability of the enzyme under acidic pH (4.2 and 5.2) conditions was assessed. Therefore, Good’s buffers (MOPS and HEPES) and common laboratory buffers, such as Tris, phosphate, and acetate, were used.

The results, which are listed in Table 2, indicate that among all the tested buffers, the enzyme displayed its highest T_m_ (65.9 ± 0.3 °C) in 20 mM NaH_2_PO_4_ (pH 8). Notably, other common buffer systems, such as HEPES, Tris, and MOPS, provided a lower level of stabilization to FosAKP. Similarly, acidic pHs (e.g., 4.2 and 5.2) were found to have a detrimental effect on enzyme stability. Next, we investigated the effect of catalytically important metals (e.g., Mn^2+^ and K^+^) on the stability of FosAKP. The results showed that Mn^2+^ provided a stabilizing effect to the enzyme, leading to an increased T_m_ value to 67.3 ± 0.1 °C. On the other hand, KCl slightly decreased the T_m_ value to 64.7 ± 0.3 °C. The presence of both metals (Mn^2+^ and K^+^) gave approximately the same T_m_ value (67.5 ± 0.2 °C) as that measured in the presence of Mn^2+^ alone.

### 2.4. Tethering of FosAKP

From a biotech perspective, the absolute substrate specificity displayed by FosAKP towards Fos is a desirable catalytic feature, as it provides the basis for its exploitation in analytical applications, such as the development of a FosAKP-based enzyme biosensor for the determination of Fos in biological samples. To develop a biosensing catalytic material, FosAKP was tethered via cross-linking with glutaraldehyde and bovine serum albumin (BSA). This tethering method can be described as a chemical method for enzyme immobilization on a three-dimensional polymer matrix (BSA) [33,34]. Enzyme molecules were not immobilized on a carrier but rather cross-linked and trapped in a matrix consisting of BSA and glutaraldehyde. Glutaraldehyde is considered one of the most appealing coupling agents in biochemistry, acting as a bifunctional reagent able to react with the primary amine groups of both BSA and the enzyme, leading to their cross-linking [33,34,35,36]. The effectiveness of glutaraldehyde as a coupling agent depends on several variables, such as the buffer system, pH, and concentration. Therefore, careful selection and consideration of the conditions are necessary to maximize immobilization efficiency [37].

**Figure 5:**
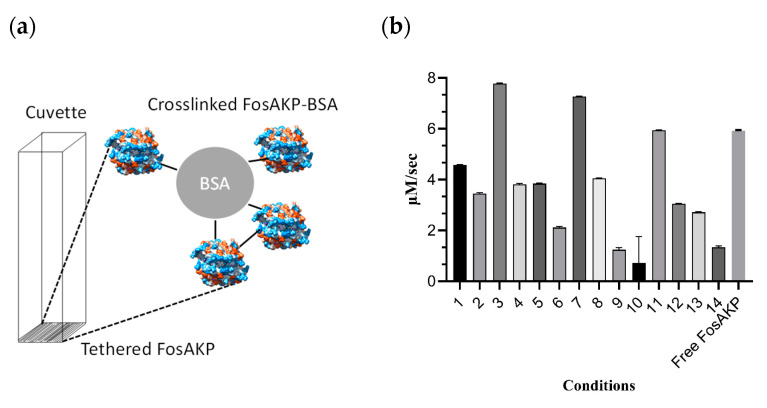
(**a**) Schematic representation of the biosensing material. FosAKP was tethered via cross-linking with glutaraldehyde and bovine serum albumin (BSA). The cross-linked proteins were deposited on the bottom of a plastic (polystyrene) cuvette. (**b**) The effect of different conditions on FosAKP immobilization yield. The conditions are shown in Table 3. Different amounts of FosAKP, buffer (50 mM sodium phosphate (pH 7)), BSA, KCl (100 mM), MnCl_2_ (0.05 mM), and glutaraldehyde were mixed. The mixture was deposited at the bottom of a cuvette (4 mL) and was left to solidify for 45 min at 25 °C, following for 24 h at 4 °C. These experiments were performed in triplicate and the enzyme activity was expressed in μΜ/s.

Various parameters were investigated to determine the optimal tethering conditions. A range of mixtures composed of different concentrations of FosAKP, MnCl_2_, KCl, and glutaraldehyde were deposited at the bottom of a 4 mL cuvette and left at 4 °C for 3 days. MnCl_2_ and KCl were added to the mixture to stabilize the enzyme structure. The results are shown in Figure 5, and Table 3 summarizes the tested tethering conditions. It was evident that conditions 3 and 7 (Table 3) displayed a higher efficiency. Between these two conditions, the latter was chosen for all further experiments, since the gel that was formed under condition 3 displayed lower stability, showing extensive deterioration after approximately 12 days. This was presumably due to the lower glutaraldehyde concentration used (0.5% *v*/*v*). Therefore, condition 7 (comprising glutaraldehyde (0.7% *v*/*v*), BSA (33 mg), and the enzyme (13.5 U)) was selected for further studies.

**Figure 6:**
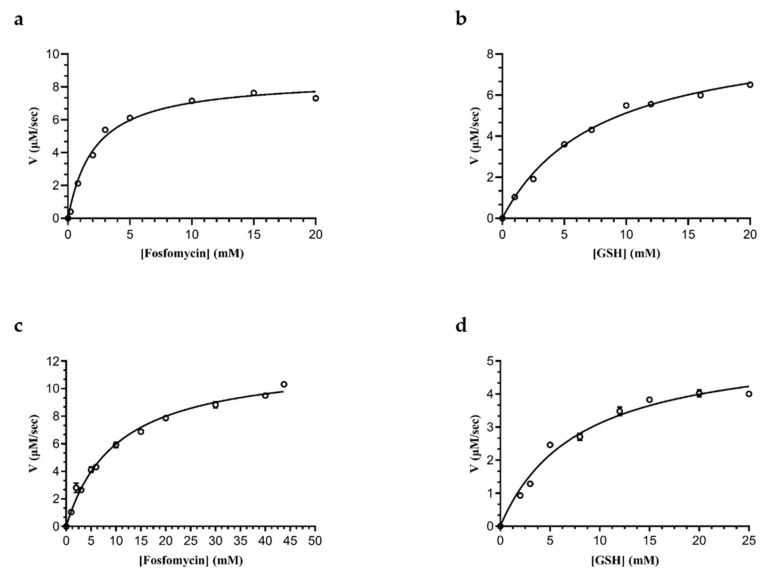
The kinetics of free (**a**,**b**) and tethered (**c**,**d**) FosAKP. (**a**,**c**) The dependence of enzyme velocity on Fos concentration using GSH at a constant concentration (15 mM). (**b**,**d**) The dependence of enzyme velocity on GSH concentration using Fos at a constant concentration (10 mM). Data are reported as the mean ± standard error of three replicates. The Michaelis–Menten equation was fitted to the data (R^2^ = 0.9915 for (**a**), R^2^ = 0.9939 for (**b**), R^2^ = 0.9857 for (**c**), and R^2^ = 0.9802 for (**d**), using GraphPad Prism 8.4.3.

Kinetic analysis of the tethered FosAKP using Fos and GSH as variable substrates was performed through steady-state kinetic measurements (Figure 6). The K_m_^FOS^ values of the free and tethered enzymes were 2.2 ± 0.3 mM and 10.2 ± 0.7 mM, respectively. The K_m_^GSH^ values of the free and tethered enzymes using GSH as a variable substrate were measured as 8.0 ± 0.8 mM and 7.9 ± 1.0 mM, respectively.

**Figure 7:**
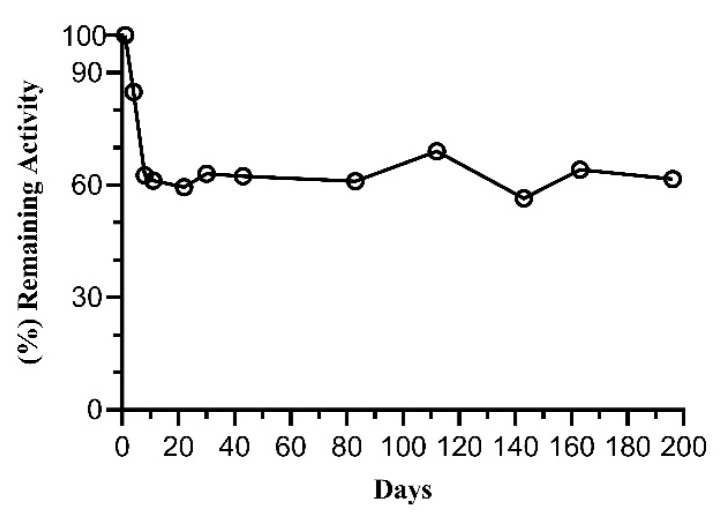
Storage stability of tethered FosAKP. The tethered FosAKP was stored at 4 °C for 196 days. At the indicated time, its enzymatic activity was measured. Datasets were analyzed using GraphPad Prism 8.4.3.

The storage stability of the tethered enzyme was studied at 4 °C (Figure 7). The results showed that the tethered enzyme retained 61.6 ± 0.03 (%) of its initial activity after 196 days. These data suggest that the glutaraldehyde chemistry used for enzyme cross-linking is suitable for FosAKP, allowing for the long-term operation of this enzyme.

### 2.5. Determination of Fos in Tap Water and Sheep Plasma Samples

**Figure 8:**
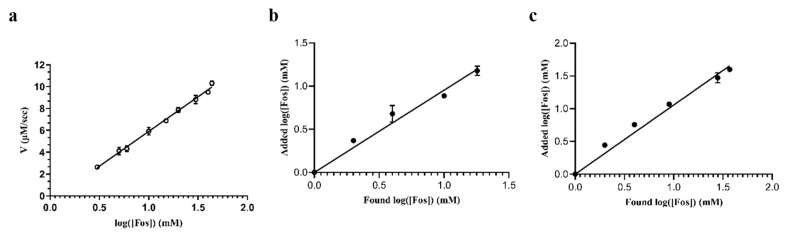
(**a**) A standard curve to demonstrate the dependence of the initial velocity of the reaction (μM/sec) on Fos concentration. (**b**) Correlation curve of added Fos concentration with found concentration in tap water using tethered FosAKP. (**c**) Correlation curve of added Fos concentration with found concentration in sheep plasma using tethered FosAKP. The datasets in the graphs were analyzed via linear regression analysis using GraphPad Prism 8.4.3.

The proof-of-concept of the suitability of tethered FosAKP was demonstrated through its application in the determination of Fos in two different matrices: tap water and sheep plasma samples. The sensing signal of the enzyme-based biosensor was based on the colorimetric assay used to measure enzymatic activity (DTNB (412 nm)), and a typical standard curve is shown in Figure 8a. The limit of detection (LOD) of the method was 1.2 mM, and the limit of quantification (LOQ) was 1.6 mM. Recovery experiments were performed using drinking tap water spiked with known amounts of Fos (2.0 mM, 4.0 mM, 10.0 mM, and 18 mM); the results are presented in Table 4. The recoveries ranged between 88.7% and 122.5%, with a mean value (*n* = 4) of 106.7%, a standard error (SE) of 9.0, and a standard deviation (SD) of 17.9. The reproducibility of this method was expressed by the (%) RSD that was calculated based on the mean values of the recoveries of Fos in the drinking water samples (16.8%).

Fos is currently used as a veterinary drug to treat infectious diseases in sheep, broiler chickens, and pigs [38]. Therefore, recovery experiments were also performed in sheep plasma spiked with Fos (2.0–37.0 mM). The results are listed in Table 5. The recoveries ranged between 101.6 % and 146.8% with a mean value (n = 5) of 115.8%, a SE of 8.3, and a SD of 18.5. The reproducibility of this method was expressed by the (%) RSD, which was calculated based on the mean values of the recoveries of Fos in the blood plasma samples (16.0%). Figure 8 shows the correlation between the added and found concentrations of Fos in tap water and spiked plasma. In both cases, a linear relationship was observed, indicating the accuracy of the proposed enzymatic biosensor for Fos concentration.

Based on the pharmacodynamics studies of Fos, the suggested dosing regimen is 6–12 g per day [19]. Owing to its low oral bioavailability (50%), Fos is only permitted for oral administration at 3 g doses (approximately 50 mg/kg body weight). The maximum serum concentrations of Fos after a 2–2.5-h oral dose of 3 g Fos correspond to a serum concentration of 21.8–32.1 mg/L, with a total area under the curve (AUC) of 145–193 mg·h/L [39]. The pharmacodynamics data suggest that the limit of detection of the FosAKP biosensor (Table 5) lies within the expected working concentration of Fos in the serum.

## 3. Materials and Methods

### 3.1. Materials

Reduced GSH and albumin (BSA (fraction V)) were obtained from Sigma-Aldrich (Sigma-Aldrich Co., St. Louis, MO, USA). Chloramphenicol was purchased from Fluka (Steinheim, Germany). Fosfomycin (disodium salt) was obtained from Apollo Scientific (Whitefield Rd, Bredbury, Stockport, UK). 5,5′-Dithio-bis(-2-Nitrobenzoate) (DTNB) was obtained from Boehringer Mannheim. Isopropyl 1-thio-β-galactopyranoside (IPTG) and ampicillin (sodium salt) were obtained from PanReac AppliChem, (Darmstadt, Germany). The KAPA HiFi PCR kit and KAPA Taq PCR kit were obtained from Kapa Biosystems (KAPA Biostystems Pty, Cape Town, South Africa). The plasmid isolation kit was purchased from Macherey-Nagel (Macherey-Nagel GmbH & Co., Düren, Germany). The In-Fusion HD Cloning Plus package (including the cloning enhancer, Infusion HD cloning kit, Clone Amp Hifi PCR Premix, and T4 DNA ligase) was obtained from Takara Bio (Takara Bio USA Inc., Mountain View, CA, USA). Yeast extract and peptone were purchased from Scharlau (Sentmenat, Spain). The protein thermal shift dye kit was obtained from Thermo Fischer Scientific (Applied Biosystems, Foster City, CA, USA).

### 3.2. Methods

#### 3.2.1. Molecular Cloning

The coding DNA sequences for FosAPA and FosAKP (accession numbers: WP_003082280.1 and WP_002887377.1 for the FosAKP and FosAKP, respectively) were optimized for the *E. coli* BL-21 expression system and synthesized by Eurofins Genomics (Abersberg, Germany). The amplification of the FosAPA and FosAKP sequences was performed via PCR using KAPA HiFi polymerase. PCR primers were designed according to codon-optimized sequences. The reactions were carried out in a final volume of 50 μL, containing 10 μM of each primer (5′-GAAGGAGATACCCTTATGCTTACCGGCCTCAATCAC-3 and 5′-GTGATGATGACCCTTGTCGGCAAAGCGCATCCC-3′ for FosAPA, and 5′-GAAGGAGATACCCTTATGTTGAGCGGTCTGAACC-3′ and 5′-GTGATGATGACCCTTCTGCTCAAAGAACACCATGCC-3′ for FosAKP), 10 pg of template DNA, 10 mM KAPA dNTP mix, 5X KAPA HiFi buffer (10 μL), and 1U KAPA HiFi polymerase. The PCR procedure consisted of an initial denaturation at 95 °C for 3 min, 30 cycles of denaturation at 98 °C for 20 s, annealing for 15 s at 67.5 °C (for FosAPA) and 66.2 °C (for FosAKP), and polymerization at 72 °C for 50 s. A final extension at 72 °C for 10 min was performed after the 30th cycle. The resulting PCR amplicons were treated with cloning enhancer buffer, according to the manufacturer’s instructions (Infusion HD cloning kit, Takara Bio USA Inc., Mountain View, CA, USA). The procedure involved the addition of 2 μL of cloning enhancer to 5 μL of each of the PCR products (linearized pEXP5-CT/TOPO vector, FosAPA amplicons, and FosAKP amplicons). The tubes were incubated at 37 °C for 20 min and then at 80 °C for 20 min. The purified products were cloned into the pEXP5-CT/TOPO vector following the procedure described in the Infusion HD cloning kit. The reaction was conducted in a total volume of 5 μL, containing 1 μL of 5X Infusion HD enzyme premix, 1 μL of linearized pEXP5-CT/TOPO vector, and 2 μL of purified PCR products (FosAPA or FosAKP amplicons). The reaction mixtures were incubated at 50 °C for 15 min. The In-Fusion reaction mixtures were then used to transform competent *E. coli* Stellar cells, according to the manufacturer’s instructions (Infusion HD cloning kit, Takara Bio). Transformed Stellar cells were spread on LB agar plates containing 100 μg/mL ampicillin and incubated at 37 °C overnight. The resulting expression constructs (pEXP5-CT/TOPOfosAPA and pEXP5-CT/TOPOfosAKP) carrying a C-terminal 6xHis tag were sequenced and used to transform chemically competent *E. coli* BL-21(DE3)pLysS cells.

#### 3.2.2. Expression and Purification FosAPA and FosAKP

*E. coli* BL-21(DE3)pLysS cells harboring recombinant plasmids (pEXP5-CT/TOPOfosAPA or pEXP5-CT/TOPOfosAKP) were grown overnight at 37 °C under shaking (180 rpm) in LB medium containing 100 μg/mL ampicillin and 33 µg/mL chloramphenicol. The cultures were used to inoculate fresh LB medium supplemented with 100 μg/mL ampicillin and 33 µg/mL chloramphenicol. The expression of the recombinant enzymes (FosAPA and FosAKP) was induced by adding isopropyl-1-thio-β-galactopyranoside (IPTG) to a final concentration of 1 mM when the absorbance at 600 nm reached 0.5–0.6, followed by incubation at 37 °C for 4 h at 180 rpm. Then, the cells were harvested via centrifugation at 8500× *g* for 10 min. The cells were resuspended in lysis buffer (50 mM Na_2_HPO_4_, 300 mM NaCl, and 10 mM imidazole (pH 8)), lyzed via ultrasonication, and centrifuged for 12 min at 13,000× *g*. The resulting supernatant was dialyzed overnight against 50 mM Na_2_HPO_4_ and 300 mM NaCl (pH 8). The purification of FosAKP or FosAPA was achieved using immobilized metal ion affinity chromatography (Ni-IDA-Sepharose) [40,41]. The dialyzed supernatants were centrifuged for 12 min at 8000× *g* and loaded onto a 1 mL column, which was previously equilibrated with lysis buffer. Non-adsorbed proteins were washed off with 10-bed volumes of lysis buffer, 8-bed volumes of wash buffer 1 (50 mM Na_2_HPO_4_ and 300 mM NaCl (pH 8)), 6-bed volumes of wash buffer 2 (50 mM Na_2_HPO_4_, 300 mM NaCl, and 20% glycerol (pH 8)), and 8-bed volumes of wash buffer 3 (50 mM Na_2_HPO_4_ and 300 mM NaCl (pH 6.3)). The bound enzymes (FosAKP or FosAPA) were eluted with elution buffers (50 mM Na_2_HPO_4_ (pH 8), containing 300 mM NaCl and different concentration of imidazole: 5 mM, 20 mM, 50 mM, 100 mM, 150 mM, 200 mM, and 250 mM). Purified FosAKP and FosAPA were eluted in the fractions containing 200 mM and 250 mM imidazole, respectively. The purity of the proteins was judged via SDS-PAGE (12.5% gel), as described by Laemmli [42].

#### 3.2.3. Assay of the Enzyme Activity and Protein Concentration Determination

FosA activity was measured spectrophotometrically by estimating the rate of GSH conjugation using 5,5-dithiobis(2-nitrobenzoic acid) (DTNB—Ellman’s reagent). The assays were performed at 25 °C, as described previously by Beharry and Palzkill [9], in 0.1 M sodium dihydrogen phosphate buffer, pH 8, containing 100 mM KCl, 0.05 mM MnCl_2_, 10 mM Fos, 15 mM GSH, and 0.25–0.50 μg of enzyme in a final assay volume of 100 μL. The reactions were allowed to run until the enzyme activity was quenched at certain time points with the addition of methanol (300 μL). In parallel, a control reaction was also carried out under identical conditions in the absence of FosA. These measurements were taken for 50–60 min until a plateau in the enzymatic reaction was reached. Plateaus were reached when a difference of approximately 0.4 was observed between the absorbance of the control and the test cuvettes. The concentration of unreacted GSH was measured at 412 nm using a UV/Vis Lambda 16 photometer (Perkin Elmer, Waltham, MA, USA) in the assay mixture composed of 0.1 M sodium dihydrogen phosphate buffer (pH 8), 1 mM EDTA, DTNB (0.0714 mg/mL), and 14.3 μL of the methanol-quenched reaction. The reactions were incubated for 15 min in the dark (25 °C), and the absorbance was recorded at 412 nm. The amount of conjugated GSH was determined by subtracting the absorbance at 412 nm from that of the control. Νon-linear regression analysis was used for the data analysis (GraphPad Prism 7.00 software program). Measurements were performed, at least, in triplicates. Catalytic assays were performed in the presence or absence of sodium Tar (0–20 mM). Before the addition of Fos, the assay solution was incubated for 10 min with Tar. Enzymatic assays of FosAPA and FosAKP using common glutathione transferase substrates (Supplementary Table S1) were carried out, as described by Georgakis et al., 2020. The protein concentration of each sample was determined using the Bradford method, using bovine serum albumin (fraction V) as the standard [43].

#### 3.2.4. Thermal Stability

The thermal stability of FosAKP was measured using differential scanning fluorimetry (DSF) on an Applied Biosystems^®^ Real-Time PCR StepOne™ instrument. Thermal stability was assessed in different buffers and additives using the Protein Thermal Shift™ dye (Applied Biosystems, Foster City, CA, USA) [44]. Fluorescence monitoring was performed at 15–99 °C at a rate of 1 °C/min. Melting temperatures (T_m_) were estimated using the GraphPad Prism 7.00 computer program by fitting the Boltzmann sigmoidal curve to the normalized data.

#### 3.2.5. Crystallization

Purified FosAKP was concentrated to 12 mg/mL in 10 mM HEPES/NaOH buffer (pH 7.0), NaCl 100 mM, and NaN_3_ 0.002% *w*/*v*. Prior to crystallization, the protein was mixed with Fos and MnCl_2_ to a final concentration of 6 mM for both of the compounds. Crystals that were suitable for X-ray data collection were grown using the hanging drop vapor diffusion method at 16 °C. The well solution consisted of 18% PEG3350 (*w*/*v*) and 0.2M potassium sodium tartrate. The well solution was mixed with an equal volume of FosAKP protein solution on siliconized cover slides.

#### 3.2.6. Structure Determination

X-ray diffraction data were collected on the BioMAX beamline at MAX IV (Lund, Sweden) using cryogenic temperatures (100 K) with 20% *v*/*v* glycerol as a cryoprotectant. Initial phases were obtained via molecular replacement using PHASER, as implemented in PHENIX [45]. The previously determined structure of FosAKP (PDB id 5V3D) was used as a search model. Refinement was performed using PHENIX 1.20 software. Model building and inspection of the structure and the electron density maps were performed with COOT 0.9.8 [46]. The data collection and final refinement statistics are presented in Table 1.

#### 3.2.7. FosAKP Tethering via Covalent Cross-Linking

FosAKP was tethered via cross-linking with glutaraldehyde and BSA. A range of different conditions were tested for establishing the optimal concentrations of FosAKP, BSA, and glutaraldehyde required for maximizing the immobilization yield. Different amounts of the enzyme, buffer (50 mM sodium phosphate (pH 7)), BSA, KCl (100 mM), MnCl_2_ (0.05 mM), and glutaraldehyde were mixed. The mixture was deposited on the bottom of a plastic cuvette (polystyrene (4 mL)) and allowed to solidify for 45 min at 25 °C, followed by 24 h at 4 °C. A control mixture containing, instead of FosAKP, the buffer (50 mM sodium phosphate dihydrogen (pH 7)) was also prepared under identical conditions. Next, 50 mM sodium phosphate dihydrogen buffer (pH 7,) was added on the top of the solidified mixtures and left at 4 °C for 3 days. The solidified gels were then rinsed (3 times) with 0.1 M sodium phosphate buffer, pH 8.0 (3 mL) to remove the excess of unreacted compounds and were stored at 4 °C until further use. The activity of the tethered FosAKP was measured as described for the free enzymes. Immobilization yield was defined as the percentage of enzyme activity (units) that was measured after the immobilization to the enzyme activity (units) that was initially added to the mixture.

#### 3.2.8. Kinetic Analysis of the Free and Tethered FosAKP

Kinetic analysis of the free and tethered FosAKP was carried out (25 °C) in 0.1 M sodium dihydrogen phosphate buffer (pH 8), containing 100 mM KCl and 0.05 mM MnCl_2_, using Fos at a constant concentration (10 mM) and variable concentrations of GSH (1.0–30.0 mM for the free enzyme and 2–25 mM for the tethered enzyme), or GSH was used at a constant concentration (15 mM) and Fos at variable concentrations (0.2–20.0 mM for the free enzyme and 1.0–43.75 mM for the tethered enzyme). The initial velocities and enzyme units were calculated as described for the free enzyme. The Michaelis–Menten model was fitted to the data using GraphPad Prism 7.00.

#### 3.2.9. Biosensor Performance and Recovery Tests

Recovery tests for Fos were performed in natural tap water (collected from Athens’s water supply network) and plasma from sheep blood (provided by the Department of Animal Science, Agricultural University of Athens), spiked with known Fos concentrations. Samples from tap water and plasma with zero Fos concentrations were used as controls. The linearity of the response was assessed from the standard curve (initial velocity of the reaction (μM/sec) versus log([Fos]) for the concentrations ranging from 1.0 mM to 43.75 mM. Linearity was observed (R^2^ = 0.9886) for the Fos concentrations between 3 mM and 43.75 mM. For measuring the concentration of Fos in the recovery experiments, the following equation was used: y = 6.348 × x − 0.451, where y corresponds to the initial velocity of the reaction (μM/sec), and x corresponds to the log([Fos]). The limit of detection (LOD) was calculated using the equation LOD = 3.3σ/S, and the limit of quantification (LOQ) was calculated using the equation LOQ = 10σ/S, where σ is the standard deviation of the response, and S is the slope of the calibration curve. Precision was assessed through the reproducibility of the method. The reproducibility of the recovery assays for each Fos concentration tested was reported as the percentage of relative standard deviation (% RSD), which was calculated based on the mean values of the recoveries of Fos in the water or blood plasma samples. Sensitivity was defined as the slope of the standard curve. Accuracy was examined measuring by calculating the percentage of recovery of Fos in the water or blood plasma samples.

#### 3.2.10. Stability Analysis of Free and Immobilized FosAKP

The stability of the tethered FosAKP upon storage at 4 °C was determined in 50 mM sodium phosphate buffer (pH 7). The enzymatic activity assays were performed as described above.

## 4. Conclusions

In the present study, soluble FosAKP and FosAPA were expressed and purified in *E. coli* BL21(DE3)pLysS. The X-ray crystal structure of FosAKP was determined at a higher resolution (1.48 Å resolution) than that reported previously. One active site of the dimeric enzyme was complexed with Fos, whereas the other site was occupied by a molecule of Tar. The binding of Tar in the active site caused slight rearrangements in the structure and dynamics of the enzyme, and consequently, Tar affects enzyme catalysis, acting as a weak inhibitor of Fos. Therefore, it is conceivable that Tar may be exploited as a lead scaffold for future drug design. The structural stability of FosAKP in different buffer systems was investigated, and the results allowed for the determination of a suitable buffer system that confers high operational stability to FosAKP. FosAKP was used to develop an optical biosensor for Fos determination, as the global problem of advancing antimicrobial resistance has led to a renewed interest in Fos use. The isoenzyme was cross-linked with glutaraldehyde to obtain a low-cost biosensor for Fos. The efficiency of the biosensor was evaluated using two different matrices, namely drinking water and sheep blood plasma. This method is simple and requires no sample preparation steps. Additionally, the biosensor displayed satisfactory recovery rates and long-term storage stability. Typically, certain issues, including the biosensor’s signal stability, reduced signal response and selectivity for in vivo analysis, shelf-life time, and bad sensor to sensor reproducibility, require further research [23,24,34]. In this case, the recovery of the biosensor could be altered when it is assessed in different samples, such as urine, since it is a complex matrix that could include substances that could interfere in the enzymatic reaction. Moreover, a correlation analysis using different matrices would also provide additional important information on the biosensor’s performance.

## Figures and Tables

**Table 1:** X-ray data processing and refinement statistics.

Data Processing	FosAKP
Beamline	BioMAX (MAX IV, Lund, Sweden)
Wavelength (Å)	0.9919
Resolution (Å)	149.2–1.48 (1.51–1.48)
Space group	*P*2_1_2_1_2_1_
Unit cell *a*, *b*, *c* (Å)	40.6, 47.0, and 149.2
No. of molecules/asymmetric unit	2
No. of unique reflections	48,410 (2174)
Completeness (%)	99.4 (92.2)
Multiplicity	9.8 (6.3)
Mosaicity (°)	0.13
*R*_meas_	0.089 (1.452)
CC_1/2_	0.999 (0.666)
Mean (I/σ(I))	11.8 (1.2)
Wilson B factor (Å^2^)	22.2
**Refinement**	
Resolution (Å)	44.85–1.48
No. of reflections used	48,242
R_cryst_/R_free_	0.173/0.204
RMSD in bonds (Å)	0.005
RMSD in angles (°)	0.899
No. of protein atoms	2203
No. of water molecules	383
Average B-factor (Å^2^)	27.7
Ramachandran favored/outliers (%)	99.6/0.0
Clashscore	4.6
PDB ID	8R37

**Table 2:** Melting temperatures (T_m_ (°C)) for FosAKP in different buffer systems in the presence or absence of additives. Melting temperatures were measured using DSF with Protein Thermal Shift™ dye. Fluorescence monitoring was carried out at 15–99 °C with a rate of 1 °C/min. Data are reported as the means ± standard errors of the means of three to nine replicates.

Buffer System	T_m_ (°C)
20 mM NaH_2_PO_4_ (pH 8.0)	65.9 ± 0.3
20 mM NaH_2_PO_4_ (pH 8.0) and 0.05 mM MnCl_2_	67.3 ± 0.1
20 mM NaH_2_PO_4_ (pH 8.0) and 100 mM KCl	64.7 ± 0.3
20 mM NaH_2_PO_4_ (pH 8.0) and 0.05 mM MnCl_2_/100 mM KCl	67.5 ± 0.2
20 mM NaH_2_PO_4_ (pH 7.0)	63.0 ± 0.2
20 mM HEPES (pH 8.0)	61.9 ± 0.2
20 mM MOPS (pH 8.0)	61.0 ± 0.2
10 mM Tris/75 mM NaCl (pH 7.5)	59.4 ± 0.1
20 mM Tris (pH 7.8)	58.7 ± 0.2
20 mM HEPES (pH 7.0)	57.6 ± 0.1
20 mM L-Arg/20 mM L-Glu (pH 7.5)	56.8 ± 0.3
10 mM acetate (pH 4.2)	53.7 ± 0.0
10 mM acetate (pH 5.2)	44.3 ± 0.0

**Table 3:** Different conditions that were tested for FosAKP immobilization. In each condition, MnCl_2_ and KCl were added in the reaction mixture at the final concentrations of 50 μΜ and 100 mM, respectively. The reactions were carried out in 50 mM NaH_2_PO_4_ buffer (pH 7).

Run Order	Glutaraldehyde (%*v*/*v*)	BSA (mg)	Enzyme (units)
1	0.5	16.5	13.5
2	0.5	16.5	6.8
3	0.5	33.0	13.5
4	0.5	33.0	6.8
5	0.7	16.5	13.5
6	0.7	16.5	6.8
7	0.7	33.0	13.5
8	0.7	33.0	6.8
9	1.0	16.5	13.5
10	1.0	16.5	6.8
11	1.0	33.0	13.5
12	1.0	33.0	6.8
13	0.7	22.0	6.8
14	1.0	22.0	6.8

**Table 4:** Fos recovery experiments in drinking tap water spiked with three known concentrations of Fos (2 mM, 4 mM, 10 mM, and 18 mM). The experiments were performed at least in triplicates and were reported as the (%) mean ± standard error. The reproducibility of the recovery assays for each Fos concentration that was tested was reported as the percentage of relative standard deviation (% RSD).

Fos Concentration (mM)	Recovery Tests (%)	Reproducibility of Recovery Tests (%)
2	122.5 ± 4.8	6.7 (n = 3)
4	121.7 ± 4.6	6.6 (n = 4)
10	88.7 ± 2.3	4.6 (n = 3)
18	93.9 ± 4.3	8.0 (n = 4)

**Table 5:** Fos recovery experiments in plasma spiked with five known concentrations (2.0 mM, 4.0 mM, 9.0 mM, 28.0 mM, and 37.0 mM). Fos recovery experiments were performed in triplicates and were reported as the (%) mean ± standard error. The reproducibility of the recovery assays for each Fos concentration that was tested was reported as the percentage of relative standard deviation (% RSD).

Fos Concentration (mM)	Recovery Tests (%)	Reproducibility of Recovery Tests (%)
2	146.8 ± 0.6	0.7 (n = 3)
4	116.8 ± 5.5	8.1(n = 3)
9	112.0 ± 1.6	2.1 (n = 3)
28	101.6 ± 5.2	7.2 (n = 3)
37	102.0 ± 0.06	0.62 (n = 3)

## Data Availability

Data are contained within the article or the Supplementary Materials.

## Supplementary Materials

The following supporting information can be downloaded at: https://www.mdpi.com/article/10.3390/ijms25010085/s1.
