# Supplementary material for: Structural Studies of Klebsiella pneumoniae Fosfomycin-Resistance Protein and Its Application for the Development of an Optical Biosensor for Fosfomycin Determination
Source: Int J Mol Sci. 2023 Dec 20;25(1):85. doi: 10.3390/ijms25010085 (PMC10779102; doi:10.3390/ijms25010085)
Supplement: Supplementary file 1 [file ijms-25-00085-s001.zip › ijms-2749821-supplementary.pdf]

Supplementary materials

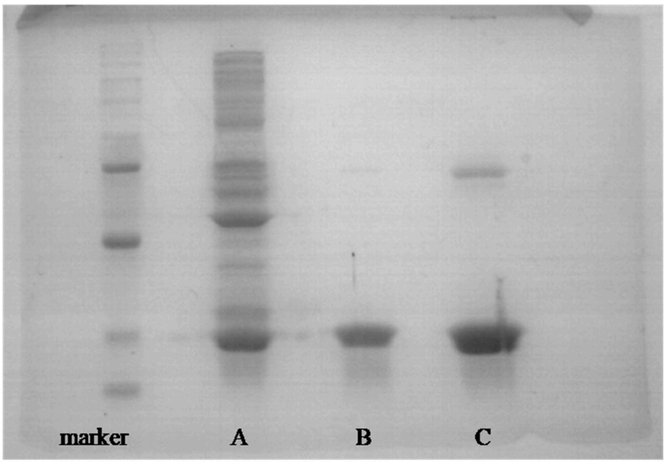

Figure S1. SDS-PAGE analysis of purified FosAKP. Purification was achieved using metal ion affinity chromatography on Ni-IDA-Sepharose column. Lane A: crude extract of *E. coli* BL-21(D3)pLysS transformed with pEXP5-CT/TOPOfosAPA vector after induction with 1 mM IPTG. Lane B: Purified FosAKP. Elution was achieved using 50 mM NaH_2PO_4 buffer containing 300 mM NaCl and 200 mM imidazole. Lane C: Purified FosAKP. Elution was achieved using 50 mM NaH_2PO_4 buffer containing 300 mM NaCl and 250 mM imidazole.

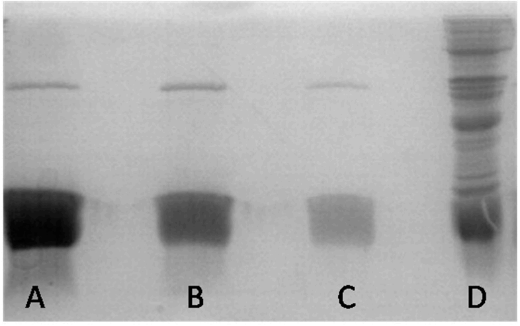

Figure S2. SDS-PAGE analysis of purified FosAPA. Purification was achieved using metal ion affinity chromatography on Ni-IDA-Sepharose column. Lane A, Lane B and Lane C: Eluted fractions of purified FosAPA. Elution was achieved using 50 mM NaH_2PO_4 buffer containing 300 mM NaCl and 250 mM imidazole. Lane D: crude extract of *E. coli* BL-21(D3)pLysS with pEXP5-CT/TOPOfosAPA after induction with 1 mM IPTG.

Table S1. Enzymatic activity of FosAPA and FosAKP using common GST substrates. Experiments were performed in triplicate.

GST Substrates	Structure	Specific Activity FosAKP (U/mg)	Specific Activity FosAPA (U/mg)
1-Chloro-2,4-dinitrobenzene (CDNB)	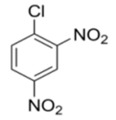	N.D.*	0.309
1-Bromo-2,4-dinitrobenzene (BDNB)	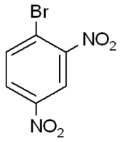	N.D.	N.D.
1-Fluoro-2,4-dinitrobenzene (FDNB)	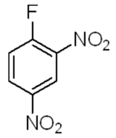	N.D.	0.020
1-Iodo-2,4-dinitrobenzene (IDNB)	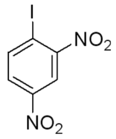	N.D	N.D.
1,2-Epoxy-3-(p-Nitrophenoxy)propane (EPNPP)	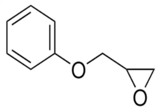	N.D	N.D.
p-Nitrobenzyl chloride (PNBC)	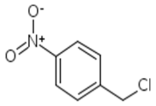	N.D.	N.D.
4-Chloro-7-nitrobenzofurazan (NBC-Chl)	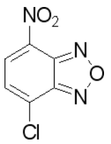	N.D	N.D.
Fluorodifen	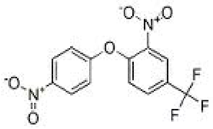	N.D	N.D.

2,3-Dichloro-4-[2-methylene-butyryl] phenoxy) acetic acid (Ethacrynic acid)	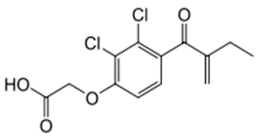	N.D.	N.D.
trans-4-Phenyl-3-buten-2-one (NSS)	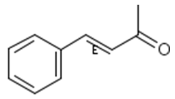	N.D.	N.D.
trans-2-Nonenal	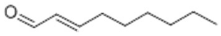	N.D.	N.D.
Allyl isothiocyanate	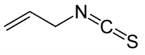	N.D.	N.D.
Phenethyl isothiocyanate (PEITH)	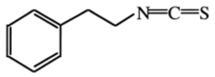	N.D.	N.D.
Dehydroascorbate (DHAR)	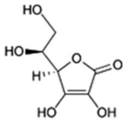	N.D.	N.D.
Bromosulfophthalein	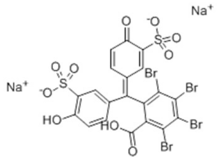	N.D.	N.D.
Cumene hydroperoxide (CuOOH)	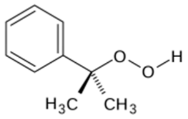	N.D.	0.444
tert-Butyl hydroperoxide	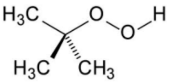	N.D.	0.066
2-Hydroxyethyl disulfide (2,2-dithiodiethanol)	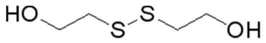	N.D.	N.D.
Sulphanilamide	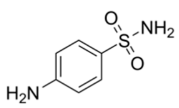	N.D.	N.D.

* No detected activity
